# Editorial: Genomic Basis of Antibiotic Resistance and Virulence in *Acinetobacter*

**DOI:** 10.3389/fmicb.2021.670975

**Published:** 2021-03-30

**Authors:** Benjamin A. Evans, Ayush Kumar, Santiago Castillo-Ramírez

**Affiliations:** ^1^Norwich Medical School, University of East Anglia, Norwich, United Kingdom; ^2^Department of Microbiology, University of Manitoba, Winnipeg, MB, Canada; ^3^Centro de Ciencias Genómicas, Universidad Nacional Autónoma de Mexico, Cuernavaca, Mexico

**Keywords:** *Acinetobacter* species, comparative genomics, antibiotic resistance, virulence, molecular epidemiology

Genome sequencing has drastically changed our understanding of the biology of many bacteria. In this respect, species from the genus *Acinetobacter* are no exception. The main goal of this Research Topic was to broaden the knowledge about antibiotic resistance and virulence for *Acinetobacter* species from a genomic point of view. This Research Topic encompasses articles adding to the biology of the genus *Acinetobacter* in three major themes: Mobiles Genetic Elements (MGE), Antibiotic Resistance, and Epidemiology and Pathogenesis.

MGEs are one of the hallmarks of the accessory genome in many bacterial species. Furthermore, they are key elements for Horizontal Gene Transfer (HGT) and known spreaders of antibiotic resistance genes (ARGs). In their contribution Leal et al. analyzed 46 *Acinetobacter* spp. isolates (all but one *A. baumannii*) from 5 hospitals in Recife, Brazil. They showed that different lineages defined by multilocus sequence typing (MLST) sequence types (STs) are spreading in hospitals in Recife. Notably, ARGs were clearly associated with different MGEs. The authors concluded that polyclonal dissemination is peppered with exchange of MGEs containing ARGs. MGEs can also be a source of genome rearrangements; in this regard, Castro-Jamies et al. showed that this is the case for the understudied *A. haemolyticus*. Analyzing 47 genomes (including 30 Mexican isolates sequenced by them), they demonstrated that this species has an open pangenome and that hypervariable regions have an array of diverse MGEs. Notably, even isolates belonging to the same clade can have large differences in gene content. Thus, it is fair to say that *A. haemolyticus* has a very dynamic genome. Plasmids are one of the most well-known mechanisms for disseminating ARGs. To gain a better understanding of plasmid evolution in *A. baumannii*, Salgado-Camargo et al. conducted an *in silico* analysis of 173 *A. baumannii* plasmids covering 47 STs and 17 countries. They found that some plasmids might have the capacity to replicate in other bacterial genera, while other groups of plasmids seem to be confined to the genus *Acinetobacter*. Moreover, some plasmids are extensively distributed in the species, notwithstanding the apparent absence of the mobilization mechanisms. They also noted that around 35% of the plasmids have ARGs and that transposons are important elements concerning gene flow between plasmids. For their part, Mindlin et al. characterized 44 plasmids from 5 permafrost *A. lwoffii* isolates to understand the diversity of plasmids in this *Acinetobacter* species. They found that a small number of plasmids are distributed in clinical settings and the environment. Remarkably, plasmids from the permafrost isolates are closely related to plasmids from clinical isolates and some of them carry ARGs in the accessory region. The authors inferred that clinical plasmids originated from the plasmids present in the permafrost. The icing on the cake is the study by Ghaly et al. They studied 21 multidrug resistance mega-plasmids (present in 11 *Acinetobacter* spp.) sampled from diverse locales across the world. They demonstrated that plasmids sampled within the same region are often distantly related in terms of the core-genome; however, they are much more similar concerning the accessory genome. Importantly, they also determined that plasmids from different geographic areas are enriched in region-specific functional capacities. Importantly, these plasmids encode 221 ARGs conferring resistance against 13 drug classes. The authors concluded that mega-plasmids are spreaders of ARGs. Collectively, the above studies clearly show that MGEs in *Acinetobacter* have a very important role in spreading multidrug resistance phenotypes. Beyond that, these and others studies suggest that MGEs in *Acinetobacter* species significantly contribute to the acquisitions of genes required for functions under different living conditions.

*Acinetobacter baumannii* displays an impressive capability to resist the action of antibiotics. It uses a combination of intrinsic and acquired mechanisms to gain antibiotic resistance. In this issue half a dozen manuscripts describe some of these resistance mechanisms. Efflux of antibiotics, mediated by RND efflux pumps, is an important means of intrinsic resistance in Gram-negative bacteria. Antibiotic resistant *A. baumannii* isolates are reported globally and this is highlighted in the collection of manuscripts in this issue. Roy et al. describe a 7-year study from India where antibiotic resistance mechanisms were evaluated in neonatal septicaemia *A. baumannii* isolates. Worryingly, they found more than 90% of the isolates were resistant to fluoroquinolones (FQ). This resistance was associated primarily with mutations in the target genes, as more than 90% of the FQ resistant isolates carried mutations in *gyrA* and *parC*. They found widespread overexpression of efflux pumps, in particular that of AdeABC. This concerning finding highlights the prevalence of FQ resistance in *A. baumannii* and also underscores the fact that multiple resistance mechanisms in combination contribute to the reduced susceptibility of *A. baumannii*. Three additional manuscripts describe mobile resistance elements in *A. baumannii*. These studies come from three different continents highlighting the global spread of *A. baumannii*. Cerezales et al. studied the mobile genetic elements in three multidrug resistant (MDR) isolates of *A. baumannii* from Bolivia. Large and diverse numbers of transposons, plasmids and resistance islands were discovered in these isolates. Alarmingly, some of these elements conferred resistance to carbapenems, antibiotics of last resort for the treatment of MDR *A. baumannii*. Ayibieke et al. report carbapenem resistant *Acinetobacter* spp. from Ghana. This study is significant since there is limited data on carbapenem resistant *Acinetobacter* from sub-Saharan Africa. The study highlights the importance of surveillance to better understand the antibiotic resistance patterns in *Acinetobacter* from hospital settings. Xanthopoulou et al. report an *A. baumannii* isolate from northern Spain that harbors *bla*_NDM−6_. A number of variants of *bla*_NDM−6_ have been reported in various pathogens including *A. baumannii*. Critically, the *bla*_NDM−6_-carrying isolates reported in this study came from a patient who traveled to northern Spain following a medical procedure carried out in Northwest Africa. This leads to the possibility that *bla*_NDM−6_-harboring *A. baumannii* are perhaps present beyond northern Spain. While the majority of studies on *Acinetobacter* spp. focus on *A. baumannii*, there is increasing appreciation of the fact that non-*A. baumannii* isolates can be quite significant in clinical settings. Two other studies in this issue touch upon the growing clinical relevance of non-*A. baumannii* isolates. Zhang et al. describe an isolate of *A. pittii* isolated from Zhejiang Province in China. This isolate carried *bla*_OXA−499_, *bla*_OXA−826_, and *bla*_ADC−221_ genes, but is susceptible to imipenem and intermediately susceptible to meropenem. They were able to isolate an imipenem-resistant mutant of this isolate under selective pressure. This allowed them to characterize the mutations in *bla*_OXA−499_ that confer resistance to carbapenems, thus showing the potential of *A. pittii* isolates to develop carbapenem resistance if they carry a *bla*_OXA−499_ gene. In a different paper, Lee et al. show that overexpression of AdeABC in clinical isolates of *A. nosocomialis* is correlated with reduced susceptibilities to tetracyclines tigecycline, omadacycline, and eravacycline. This study further highlights the importance of efflux pumps in reduced susceptibility of bacteria such as *A. nosocomialis* to novel antibiotics.

One of the key components that contributes to the success of *A. baumannii* as an antibiotic-resistant pathogen is its complement of outer membrane proteins. In their mini-review, Uppalapati et al. discuss the latest research on the roles of the outer membrane proteins OmpA, CarO, and OprD as virulence factors and in altering membrane permeability in *A. baumannii*. They conclude that the evidence to date suggests a major role for outer membrane proteins in host interaction, infection, and surviving antibiotic exposure, but that significant gaps in our knowledge remain. In addition to the outer membrane, the capsule of *A. baumannii* is thought to be important for infection. To investigate this, Loraine et al. examined nearly 200 clinical isolates from three hospitals in Thailand. While isolates belonging to International Clone (IC) 2 predominated, a large number of isolates from very diverse STs were identified. Intriguingly, they found a correlation between the thickness of capsule being produced and serum resistance but were not able to determine the reason for the differences in capsule production between strains. This highlights the potential role of the capsule in infections with *A. baumannii* but shows that there is still much to learn about capsule variability, production, and function. Understanding the variation between the major clonal lineages of *A. baumannii*, and the implications of this, are a major goal of current research. To this end, Mancilla-Rojano et al. carried out a study of *Acinetobacter* isolates collected between 2015 and 2017 from a children's hospital in Mexico. A high proportion of the isolates were either MDR (44%) or extensively drug resistant (11%) and were spread across a diverse range of 27 different STs. Interestingly, they report nine instances of more than one ST causing infection in the same patient. Clonal complex (CC) 79 was the most prevalent (27% of isolates), which belongs to IC5. It has recently become apparent that the major epidemic lineages in Latin America are not IC1 and IC2, but are IC4, IC5, and IC7. There are very few detailed studies focussing on these strains, and therefore the study by Nodari et al. looked to address this. They studied carbapenem resistant *A. baumannii* isolates from five different Brazilian hospitals, and found isolates belonged to either IC4 or IC5. All isolates were MDR including high levels of resistance to polymyxins (72%) and carried genes for the carbapenemases OXA-23 or OXA-72. These studies highlight the importance of International clones other than IC1 and IC2 in Latin America, which is an area that deserves far more attention. The global prevalence of carbapenem-resistant clonal lineages has made strategies to combat these a priority. This was the goal of Chen et al. who show the value of a long-term study on the impact of a comprehensive intervention designed to reduce the burden of *A. baumannii* in hospitals. The intervention was successful in reducing overall resistance rates, and the frequency and duration of outbreaks. However, levels of resistance in the imipenem-resistant isolates did not decrease, except for tigecycline. The prevalent OXA-58 carbapenemase-encoding isolates disappeared following the intervention and were replaced with isolates encoding OXA-23. While it is encouraging that such intervention strategies can be effective, their impact upon the population structure of *A. baumannii* and associated resistance and virulence phenotypes is still not clear.

The advent of affordable genomics has greatly increased our understanding of the factors influencing antibiotic resistance and virulence in *Acinetobacter* but significant gaps in our knowledge still remain. Large-scale genomic studies, in particular with unbiased isolate selection and including currently under-represented areas of the word, will reveal the true variation within species in the genus. By combining genomics with high-quality clinical and epidemiological data, and laboratory-based assays, we will achieve a far greater understanding of the link between genotype and phenotype in these species, shedding light on the factors that have resulted in *Acinetobacter* being amongst the most feared nosocomial pathogens.

## Author Contributions

BE, AK, and SC-R drafted and edited the manuscript. All authors approved the final version.

## Conflict of Interest

The authors declare that the research was conducted in the absence of any commercial or financial relationships that could be construed as a potential conflict of interest.

